# Dinner to Dr. George B. Wood

**Published:** 1860-07

**Authors:** 


DINNER TO DR. GEORGE B. WOOD.—A complimentary dinner was given, on the sixth of May, to Dr. Wood, at the Academy of Music, at which a large number of his professional friends and admirers were present. The object of this mark of respect was to testify the regard in which he is held as a gentleman, and the estimate placed upon him as a distinguished teacher and author.

